# Interaction of Myopic Optic Neuropathy (MON) and Glaucomatous Optic Neuropathy (GON): Pathophysiology and Clinical Implications

**DOI:** 10.3390/jcm15031065

**Published:** 2026-01-29

**Authors:** Etsuo Chihara

**Affiliations:** 1 Sensho-kai Eye Institute, Uji 611-0043, Kyoto, Japan; chiha492001@gmail.com; Tel.: +81-774-45-2060; 2 Department of Ophthalmology, Faculty of Medicine, Shimane University, Enya, Izumo 693-8501, Shimane, Japan

**Keywords:** myopia, glaucoma, retinal nerve fiver, optic disc, axonal transport, myopic optic neuropathy (MON), glaucomatous optic neuropathy (GON), lamina cribrosa, tilting, central visual field defect, parapapillary choroidal atrophy

## Abstract

**Objective:** To clarify the pathophysiology of myopic optic neuropathy (MON) and its relationship to glaucomatous optic neuropathy (GON). **Background:** MON is presumed to be associated with posterior pole ectasia and deformation of the lamina cribrosa (LC) and parapapillary region. Its dependance on intraocular pressure is expected to be weaker than that of GON; however, the characteristics and clinical behavior of MON remain incompletely understood. **Methods:** A PubMed search using the keywords myopia, glaucoma, retinal nerve fiber, optic disc, and axonal transport identified 234 relevant publications, which were analyzed in this narrative review. **Results:** In myopic eyes, a large optic disc, thin or defective LC, and parapapillary microvasculature dropout (pMvD) are considered signs of increased vulnerability to glaucomatous injury. Despite these structural risk factors, visual field (VF) progression in myopic patients with glaucoma is often slow. The involvement of MON, which likely develops in young adulthood and stabilizes with aging, may explain this discrepancy. MON may substantially contribute to the development of central VF defects in myopic glaucoma, which are associated with elongation of papillomacular bundle, pMvD, and normal tension glaucoma. Experimental studies demonstrating impaired axonal transport at the optic disc margin provide important insights into the pathogenesis of MON. Additionally, optic disc deformations in myopia including disc tilting, rotation, and focal thinning or defects of the LC may contribute to atypical VF defects and altered susceptibility to glaucomatous damage. **Conclusions:** Interaction between MON and GON may explain atypical VF defects and the relatively slow VF progression observed in myopic patients with glaucoma-like VF defects.

## 1. Introduction

Myopia, especially high myopia, is a well-established risk factor for the development of open-angle glaucoma (OAG). In typical primary open angle glaucoma (POAG), early visual field defects (VFD) are characterized by Bjerrum scotomas, nasal steps, and paracentral scotomas that usually progress from the midperiphery toward central fixation. In contrast, when glaucoma coexists with high myopia, central VFD, and temporal VFDs, which are unusual in non-myopic glaucoma eyes, may appear at an early stage of the disease [1,2,3,4]. In addition, the distribution of retinal nerve fiber layer defects (RNFLDs) is often atypical compared with that seen in non-myopic glaucomatous eyes [5].

Other than the glaucomatous VFDs, different types of VFDs, named non-glaucomatous VFDs, may be observed in highly myopic eyes [6,7,8]. Why such defects occur, and whether they are related to glaucoma, are central questions addressed in this review.

In the 1970s, it was demonstrated that neural damage in glaucomatous eyes occurs predominantly at the superior and inferior regions of the lamina cribrosa (LC) [9,10]. However, impairment of axonal transport is not confined to the LC; it has also been shown to occur at sites where axons bend and are subjected to stretching stress, such as at the edge of Elschnig’s scleral ring [11,12,13]. In recent years, the concept of *myopic optic neuropathy* (MON), which is distinct from *glaucomatous optic neuropathy* (GON), has gained increasing acceptance [7,14,15,16,17,18,19]. The mechanism of neural damage in MON is thought to be associated with stretching tension of the nerve fibers and deformation of the optic disc. Because the underlying pathophysiology differs fundamentally from that of GON, the patterns of VFDs in eyes with high myopia and glaucoma-suspect differ from the typical patterns observed in GON [7,8].

When glaucoma occurs in myopic eyes, these eyes may exhibit features of both MON and GON. In addition to MON-related changes, GON in myopic eyes may be further modified by optic disc abnormalities such as optic disc tilting and rotation, reduced peripapillary vessel density, and focal thinning and/or defects of the LC. Nerve fiber damage at the LC may be exacerbated by concomitant stretching injury to the nerve fiber layer (NFL) associated with elongation of the papillomacular distance, as well as by abnormal elevation or overhanging of the scleral ridge in myopic eyes [20]. Although earlier reports of axonal transport impairment at the optic disc margin were not widely recognized, this concept may provide important insights into the pathophysiology of neural damage in MON, in which elongation of the papillomacular bundle is considered a key risk factor [21].

## 2. Literature Search Methods

Publications related to MON and myopic glaucoma were searched in PubMed. Using the keywords of myopia, glaucoma, retinal nerve fiber, optic disc, and axonal transport, a total of 512 publications were identified.

The retrieved articles were reviewed after being categorized into three groups:

(1) Basic experimental studies;
(2) Studies examining the relationship between visual field progression and myopia,
(3) Studies investigating the association between optic disc deformation and patterns of visual field impairment.

After screening for relevance, 234 publications were selected and analyzed in this narrative review.

## 3. Association Between MON and GON

Myopic eyes tend to have higher intraocular pressure (IOP) [22,23,24], larger optic nerve head size [25], a thinner LC [26], and lower corneal hysteresis [27]. These characteristics suggest increased vulnerability of the nerve fibers in myopic eyes. Accordingly, myopia is considered a risk factor for the development of GON [22,28,29,30,31,32,33,34,35,36,37,38,39,40,41,42]. GON may therefore be more prevalent in myopic eyes because retinal NFL damage may occur even at normal IOP in eyes with thin and deformed LC.

Central VFDs are common in eyes with concomitant glaucoma and myopia [1,2,3]. As discussed later in Section 7, many studies suggested that the mechanisms underlying central visual field loss include elongation and mechanical stretching of the papillomacular bundle and enlargement of the γ-zone due to posterior pole expansion of the eyeball [43], as well as the development of parapapillary choroidal atrophy (PPA) accompanied by microvascular dropout (MvD) [44]. Li et al. reported that predictors of myopic visual field defects included longer axial length (*p* = 0.026), thinner central corneal thickness (*p* = 0.013), worse baseline VF status (*p* = 0.004), and a larger γ zone (*p* < 0.001), whereas IOP was not a significant risk factor for myopic VF progression (*p* = 0.206) [7]. If elongation of the papillomacular bundle or development of MvD contribute to myopic central VFDs, the underlying pathophysiology may be attributed to IOP independent deformation of the eye. In such case, the onset of myopic VFDs would be expected to coincide with a period of progressive axial elongation of the eyeball. In this regard, several interesting studies have been published, reporting rapid progression of VFDs during young adulthood followed by stabilization at older ages [45,46,47]. This pattern contrasts with that observed in typical GON, in which nerve damage is more prevalent and progresses more rapidly in older individuals [48,49,50,51,52,53,54]. Interestingly, parafoveal scotomas have been reported to be associated with lower IOP (≦16 mmHg), myopia, and Caucasian ethnicity [55], whereas, other studies have demonstrated a positive association between IOP and VF progression in myopic normal tension glaucoma (NTG) eyes [47]. Yoshida et al. further demonstrated that substantial IOP reduction is beneficial for preserving the central visual field [56]. It may be speculated that following IOP reduction, the LC may shift anteriorly, thereby shortening the distance between the Bruch’s membrane opening (BMO) and the LC, as well as between the fovea and the LC. This anterior displacement of the LC may ultimately reduce elongation-related stretching tension on the retinal nerve fibers [57]. If MON and GON act in concert to damage the retinal NFL, IOP reduction may effectively mitigate GON-related injury and thereby contribute to preservation of the central visual field. A randomized control study evaluating the effect of IOP reduction on MON is planned; however, to the best of the author’s knowledge, no confirmatory reports have been published on this topic to date [58].

From a clinical perspective, it is often difficult to determine the primary cause of visual field abnormalities among potential contributors, including MON, GON, and myopic chorioretinal lesions. Myopic eyes often exhibit optic disc deformation, making it challenging to distinguish myopic optic disc abnormalities from GON [14,59,60,61,62,63,64,65,66,67,68,69,70,71,72,73,74,75]. Although attempts have been made to differentiate GON from myopic optic disc deformation using optical coherence tomography (OCT) and artificial intelligence, this distinction remains challenging [67,74]. Differentiation between MON and GON is clinically more difficult. However, by understanding the characteristic features of MON and GON, it may be possible to identify eyes in which either MON or GON is the dominant mechanism of optic nerve damage. If MON is relatively insensitive to elevated IOP, the therapeutic response to IOP-lowering treatment may be limited. The IOP-independent nature of MON may help explain the slow progression of VFDs observed in some eyes with high myopia and coexisting glaucoma [76].

## 4. Rationale for Considering Non-Glaucomatous Nerve Damage: Evidence from Experimental Studies

**Figure 1:**
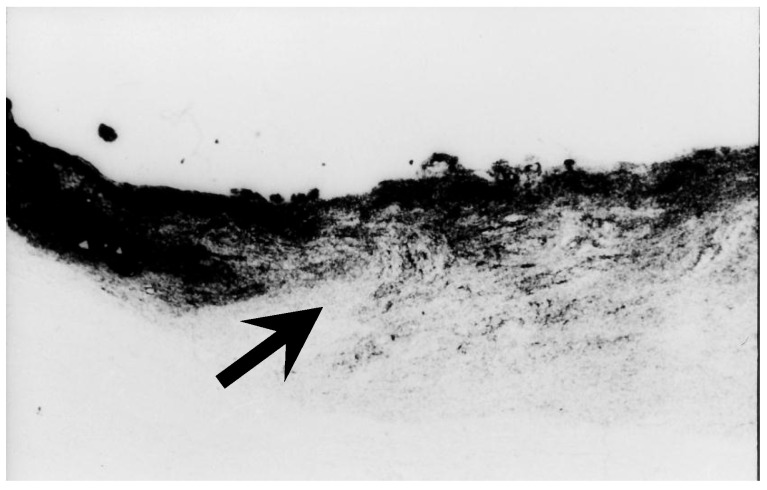
Autoradiographic evidence of axonal transport blockade at the scleral margin (arrow) in an ocular hypertensive rabbit eye. The LC is absent or poorly developed in the rabbit optic nerve head. In this autoradiography, only ^3^ H-leucine radiolabeled proteins are visualized as black signals. In ocular hypertensive rabbits, accumulation of axonally transported radiolabeled proteins is observed at the margin of the optic disc (arrow), where nerve fibers are bent, stretched, and compressed against the sclera. Radiolabeled proteins are abundant at the surface of the disc but markedly decrease in their deeper portions, indicating a blockade of axonal transport at the disc margin (Reproduced with permission from Chihara E et al. [12]).

**Figure 2:**
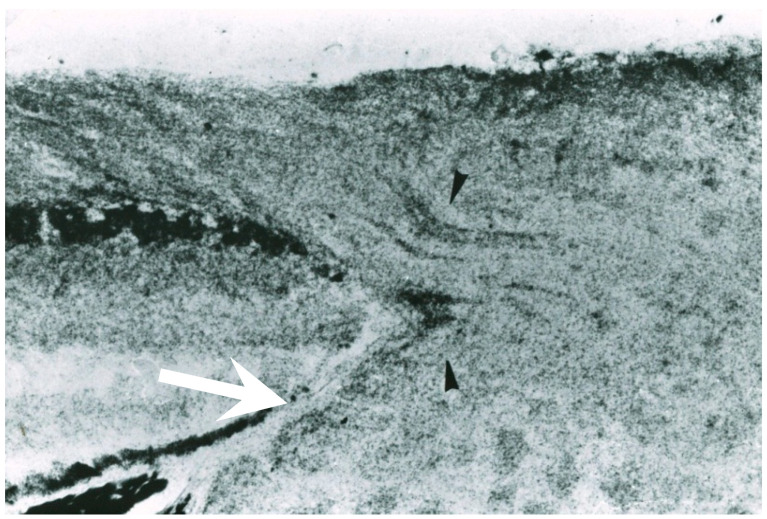
Dual blockage of axonal transport in monkey eye. In addition to the blockage of axonal transport at the LC (white arrow), axonal transport is also interrupted at the margin of the optic disc near Elschnig’s scleral ring (arrow heads). This dual-site blockage is of particular interest for explaining damage to the papillmacular bundle and central VFDs in eyes with an elongated papillomacular distance, as is observed in high myopia eyes (Reproduced with permission from Chihara E et al. [11]).

As was mentioned above, blockage of axonal transport can occur outside the LC (Figure 1). In ocular hypertensive rabbit eye, in which the LC is absent or poorly developed, accumulation of axonally transported materials has been demonstrated at the margin of the optic disc [12]. In another autoradiographic study using monkey eyes, axonal transport blockage was observed not only at the LC but also at the margin of the Elschnig’s scleral ring [11] (Figure 2). These experimental findings provide important insight into the pathogenesis of MON.

**Figure 3:**
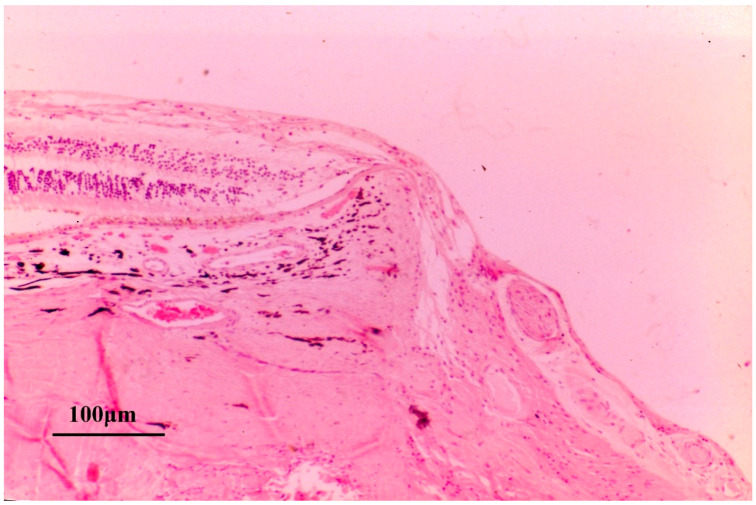
Compression of the retinal nerve fiber layer at the site of an elevated scleral ridge in an end-stage human glaucomatous eye (hematoxylin-eosin staining; scale bar = 100 µm). This specimen demonstrates a markedly elevated scleral ridge associated with severe thinning of the retinal nerve fiber layer. Because axonal transport may be impaired at the edge of the Elschnig’s scleral ring (Figure 1 and Figure 2), the combination papillomacular bundle elongation (stretching) and scleral ridge elevation may exacerbate axonal injury (Reproduced with permission from Chihara E. [83]).

In high myopia, the γ zone is widened, and the papillomacular nerve fiber bundle becomes elongated and stretched as a result of posterior globe ectasia. In addition, the parapapillary scleral ridge may be elevated [17,77,78,79]. Localized elevation of ridge-like peripapillary sclera has been reported in highly myopic eyes [79,80], and this may become associated with dome-shaped macula [81]. Such focal elevation of collagenous sclera may induce bending and stretching of the overlying retinal NFL, thereby predisposing it to nerve damage (Figure 3).

Furthermore, overhang of border tissues beyond the clinical disc margin [20] and reduced posterior scleral stiffness may also contribute to the development of RNFLDs [82].

## 5. Is Myopia a Risk Factor for Visual Field Progression?

As mentioned above, numerous studies have reported that myopia is a significant risk factor for the development of OAG [22,28,29,30,31,32,33,34,35,36,37,38,39,40,41,42,84,85], POAG [30,31,32,34,35,36,41,42,86,87,88,89], and NTG [29]. Ocular hypertensive patients with myopia also have an increased risk of developing POAG [54,90]. Many reports further indicate that VFDs and RNFL damage tend to progress more readily in myopic glaucoma eyes [4,50,53,91,92,93,94,95,96]. In addition, myopia combined with IOP fluctuation has been identified as a risk factor for NTG [94].

Several investigators have reported that myopia accelerates visual field progression in glaucoma eyes [54,90,91,92] or is associated with more rapid progress of neuronal damage in myopic eyes [4,50,93,95,96,97,98,99]. However, between 2010 and 2020, many studies reported a negative association between myopia and VF progression in NTG and OAG eyes [51,100,101,102,103], as well as POAG eyes [102,104,105,106,107], in both the central and peripheral VFDs [103]. Several investigators even suggested that myopia may be protective against VF progression in OAG [101,107].

This apparent contradiction creates a paradox: VF progression appears to be slow in many myopic eyes despite the presence of thin LC, large optic disc size, and microvascular defects, all of which suggest increased vulnerability of the NFL, as well as the well-known high prevalence of glaucoma in myopic populations.

Lee raised an important question of whether eyes with extremely slow VF progression are truly glaucomatous or not [108]. One paper attempted to explain this paradox by attributing it to defects of the LC that might confer a neuroprotective effect [109]; however, this explanation seems unconvincing, as not all myopic eyes exhibit lamina cribrosa defects (LCDs). As discussed previously, some of these slowly progressive cases may instead reflect features of MON rather than typical GON.

**Figure 4:**
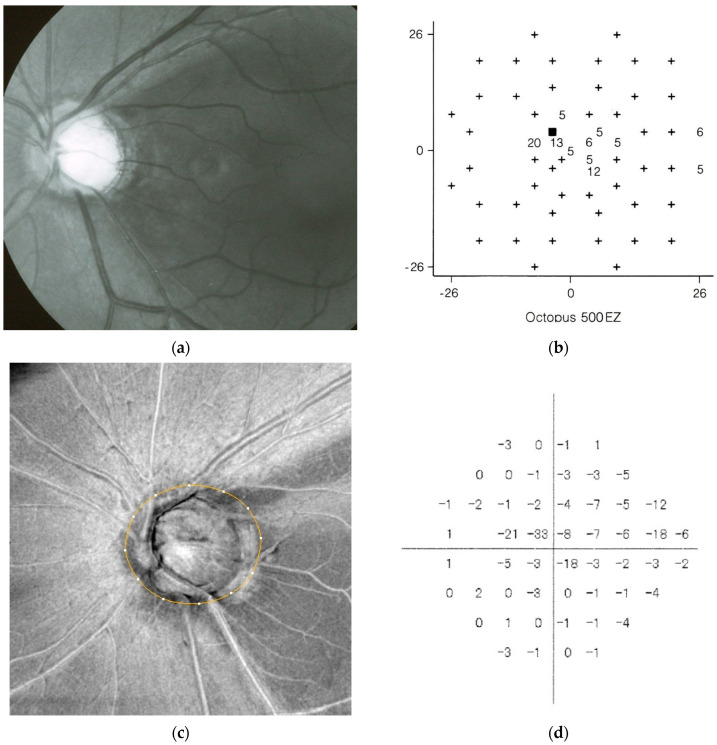
(**a**,**b**): Red free light image of papillomacular bundle defects in eyes diagnosed as so-called “normal tension glaucoma” [1], originally published in 1992 (Reproduction with permission from Chihara E et al. [1]). (**c**,**d**): Thirty-eight years after the initial examination, she was re-examined in 2025 at the age of 85 years. The papillomacular bundle defects examined using en face OCT imaging and the central visual field loss assessed by Humphrey Visual Field Analyzer were remarkably similar to those observed in 1987 (**a**–**d**). She had undergone phacoemulsification with intraocular lens implantation in 2009, after which her IOP remained in the high-teen range. In 2025, only mild VF progression was detected, characterized by an increase in the superior nasal step to −18 dB on Humphrey Field Analyzer testing; the calculated mean deviation slope was −0.11 dB/year (**d**). The yellow circle indicates the margin of the BMO. Based on these findings, this eye was diagnosed as having a combination of MON and GON, with MON-dominant RNFLDs.

MON may be largely independent of IOP. Interestingly, VF progression in myopic NTG has been reported to occur before the age of 40 [47] and stabilize after 50 years of age [45,46]. Myopic globe enlargement and progressive optic disc deformation occur predominantly during adolescence. If MON is associated with elongation of papillomacular bundle or deformation of the optic disc and parapapillary structures, it is reasonable to consider that MON-related, non-glaucomatous RNFL defect, which arises outside the LC [11,12], would develop at a younger age and subsequently stabilize once myopic globe ectasia ceases. An illustrative example of such a case is shown in Figure 4, which was reported in 1992 [1]. In this study of 117 patients, long axial length (*p* < 0.01), a diagnosis of NTG (*p* < 0.05), and a large optic disc (*p* < 0.05) were identified as significant risk factors for diffuse type papillomacular bundle defects (Figure 4).

This red free light photograph shows diffuse papillomacular bundle defects in a 45-year-old woman with high myopia (−8D) and a large optic disc (disc area 3.13 mm^2^). At the initial visit, her best corrected visual acuity in the left eye was 20/25, and IOP was 19 mmHg. Her father and elder brother had OAG. She was diagnosed with bilateral “normal tension glaucoma” with central VFDs (Figure 4b). The Octopus G1 program demonstrated a central scotoma with sensitivities reduced by 5–20 decibels (dB). The peripheral visual field was largely preserved. She was sporadically treated with a topical beta blocker for 30 years, during which her IOP ranged from 16 to 19 mmHg.

## 6. Myopic Deformations of the Optic Disc and Its Association with Visual Functions

In recent years, the concept of MON, which is distinct from GON, has gained increasing acceptance [7,14,15,16,17,18,19,110]. If retinal nerve fiber damage is predominantly associated with MON, progression of RNFLDs may be slower in older patients. This concept may help explain the paradoxically slow visual field progression observed in eyes with coexisting myopia and glaucoma.

Elongation of the papillomacular bundle in myopic eyes is one factor that may contribute to MON; however, it is not the sole mechanism of nerve damage. Deformation of the optic disc may cause local distortion, kinking, and compression of the nerve fibers, thereby increasing their vulnerability to IOP-related stress. This section focusses on the association between optic disc deformation and retinal nerve fiber vulnerability.

### 6.1. Optic Disc Morphology

#### 6.1.1. Optic Disc Size

Optic disc size in myopic eyes shows considerable interindividual variability [111,112], and several studies have reported a positive correlation between optic disc size and axial length [25,113]. However, the two-dimensional disc area tends to be smaller in eyes with optic disc tilting [112]. Both neural rim area and cup area increase with enlargement of the optic disc; however, enlargement of the cup predominates over that of the neural rim. As a result, the cup-to-disc ratio increases as disc size enlarges [114]. In addition, IOP-dependent deformation of the LC is greater in eyes with large optic discs than in those with small discs, which may explain why large discs are more vulnerable to elevated IOP and development of glaucoma.

#### 6.1.2. Effects of Optic Disc Tilting on Nerve Damage

Optic disc tilting is observed in between 0.36% and 3.5% of the general population and is frequently associated with myopia [112,115,116,117]. The presence of optic disc tilting hampers the differential diagnosis of glaucoma [118].

Both tilting and disc torsion are associated with the location of RNFLD and VFDs [119,120,121]. In particular, inferior RNFLDs are commonly observed in eyes with optic disc tilting [122,123].

##### Association Between Optic Disc Tilting and VFDs

VF progression is reported unlikely once VF progression is terminated at the region associated with optic disc tilt [124]. Although VF progression in NTG is generally slow, progression appears to be accelerated in eyes with optic disc tilting [46].

Optic disc tilt angle and the presence of β PPA are associated with myopic NTG [125]. Several studies have reported a positive association between optic disc tilting and central VFDs [126,127]. In a cohort of 960 young patients aged 26.6 years old examined for glaucoma in a refractive surgery clinic, 26 eyes were diagnosed with glaucoma. Among them, 18 eyes (69.2%) exhibited optic disc tilting [128].

Several studies have reported a positive association between optic disc tilting and faster VF progression and VF loss in eyes with NTG and OAG [129,130]. In contrast, other reports have suggested a protective effect of optic disc tilting on visual field progression [76], or a slower rate of visual field progression in eyes with a tilted optic disc [76,131,132].

##### Direction of Optic Disc Tilting and VFDs

Temporal tilting has been identified as a risk factor for VF progression in NTG [46], vertical and horizontal tilting exert different effects on retinal NFL thickness, and inferior tilting is associated with inferior NFL defects [133], as well as more advanced VF loss [119].

There is general agreement that eyes with optic disc tilting exhibit visual field patterns that differ from those observed in eyes without tilting. However, whether optic disc tilting accelerates visual field progression remains controversial, as published reports are conflicting and the available evidence is inconsistent and inconclusive.

#### 6.1.3. Ovality Index

The ovality index is correlated with optic disc tilting and is known to increase in eyes with a longer axial length [134]. It has also been shown that, as children become more myopic, both the development of β-parapapillary atrophy (β-PPA) and an increase in the ovality index occur concurrently [135,136].

#### 6.1.4. Torsion or Rotation of the Optic Disc

Cyclotorsion of the disc is associated with a larger optic disc size, longer axial length, and a shorter disc–foveal distance [112]. Its association with the location of VFDs has been discussed in several studies [137,138]. Both the prevalence and degree of optic disc torsion are significantly greater in eyes with VFDs than in those with normal visual field [139]. In myopic NTG, the direction of ONH tilting and torsion has been shown to be significantly associated with the location of VFDs [140,141].

However, the location of RNFL thinning cannot be adequately explained by disc rotation alone and appears to be more closely related to optic disc tilting [133]. Optic disc rotation not only affects optic disc morphology but is also associated with scleral thinning [142]. Like optic disc tilting, disc rotation has been reported to correlate with the location of central VFDs and RNFLDs [122,127]. In contrast to tilting, many studies have suggested that optic disc rotation is a risk factor for visual field progression [143,144].

#### 6.1.5. Congenital Anomalies, Hypoplasia, and High Myopia

The optic disc in myopic eyes is generally large; however, hypoplastic optic discs are occasionally observed [145,146,147]. Tilted disc syndrome is considered a congenital anomaly resulting from delayed closure of the embryonic fissure. It is characterized by bilateral inferonasal disc tilting, situs inversus of the retinal vessels, and bitemporal superior VFDs, and is frequently associated with posterior pole anomalies such as inferior crescent and inferior staphyloma. Tilted disc syndrome and chorioretinal coloboma may coexist with high myopia and can produce atypical VFDs [148,149,150]. In some cases, distinguishing MON from tilted disc syndrome may pose a clinical challenge.

## 7. Special Type of VFD (Central Visual FIELD Defects) and Associated Factors

Papillomacular retinal NFL defects were first reported in 1992 in eyes with long axial length, NTG, and eyes with large optic discs [1]. Subsequent studies confirmed an association between central VFD, myopia, and NTG [2,151,152], as well as between central VFD, myopia, and POAG [3,153].

In addition to myopia, optic disc rotation and tilting, disc hemorrhage, and nasal displacement of the central retinal vessel trunk have been associated with parafoveal scotomas [55,126,127,154], whereas a large PPA and LCDs have been linked to papillomacular bundle defects [155]. Temporal optic disc tilting also has been associated with central VFDs in POAG; however, the degree of tilting in these cases is generally mild [156].

Eyes with high myopia and OAG have a higher risk of developing central VFDs compared with eyes with low-to-moderate myopia [157,158]. Flattening of the optic nerve head, thinning of the prelaminar tissue, and enlargement of the γ-zone suggest mechanical stretching of the optic disc and are associated with central visual field scotoma [43,159].

Several reports further suggest that MvD or reduced vessel density in the deep parapapillary region is associated with central VFD [44,160,161,162].

Moreover, a higher degree of myopia is associated with a faster rate of visual acuity loss [4].

## 8. Elongation of Papillomacular Distance

In myopic eyes, the disc–fovea distance is elongated [112,163,164,165]. As described above, stretching of the retinal NFL is thought to induce mechanical stress and has been hypothesized to contribute to the development of MON, including central visual filed defects.

## 9. Abnormal Lamina Cribrosa (LC) and Cup of the Disc

### 9.1. Lamina Cribrosa Defects (LCDs)

When scleral ectasia occurs, pit-like scleral clefts are observed in 16.2% of eyes with high myopia [166]. This kind of ectasia-related collagenous defect may also be present in the LC. LCDs have been reported to be associated with POAG, vertical optic disc tilt, and peripapillary intrachoroidal cavitation (ICC) [167], as well as with reduced peripapillary vessel density [168]. In addition, LCDs have been linked to visual field abnormalities [169,170,171] and an increased risk of developing glaucoma [172]. Paradoxically, several studies have suggested that LCDs may exert a protective effect against further nerve damage and are not associated with progression of VFDs [109,130,173].

### 9.2. Thin LC

Enlargement of the optic disc or stretching due to axial elongation of the eye can result in thinning of the LC. High myopia is consistently associated with a thinner LC [26,174,175,176].

### 9.3. Excavation and LC Depth

The depth of the optic cup is generally shallow in non-glaucomatous eyes with high myopia [165,177]. Increased stretching tension associated with axial elongation in high myopia may contribute to this shallower excavation in non-glaucoma eyes. However, thin and structurally weakened LC in highly myopic eyes may lead to pronounced and pit-like posterior deviation in glaucoma eyes. Such increased LC depth has been reported predominantly in eyes with a long axial length subgroup [178].

Anterior LC insertion depth is not related to axial length [179]. Although overall LC depth does not differ significantly between high-myopic and non-high-myopic eyes, LC tilt is negatively associated with high myopia. As a result, the temporal or inferior portion of the LC lies closer to the reference plane [180]. In addition, a more flexible LC in younger patients tends to show greater posterior displacement than the solid LC observed in old patients [181].

## 10. Parapapillary Changes

### 10.1. Bruch’s Membrane Opening (BMO)

Enlargement and temporal shift of the BMO are common in highly myopic eyes [77,182,183,184,185,186], particularly when the axial length exceeds 26.0 mm [187]. In addition, tilting and rotation of the BMO are characteristic features of myopic eyes [188]. The BMO/LC offset is an important clinical marker for the assessment of glaucomatous damage [189].

### 10.2. Intrachoroidal Cavitation (ICC)

ICC is a large hyporeflective space located beneath the normal plane of the retinal pigment epithelium [190,191], and is associated with both myopia and glaucoma [192]. Peripapillary ICC alone does not cause corresponding VF defects; however, the presence of a full-thickness retinal defect and circumpapillary RNFLT thinning at the site of peripapillary ICC is associated with VF defects [193].

### 10.3. Parapapillary Choroidal Atrophy (PPA)

The PPA is subclassified into zones α, β, γ, and δ.

The α zone is characterized by irregular hypo- and hyperpigmentation of the Bruch membrane and retinal pigment epithelium. It is present in almost all normal eyes and is preferentially located in the temporal sector of the optic disc [194].

The β zone is characterized by the presence of Bruch’s membrane and absence of retinal pigment epithelium, with visible large choroidal vessels and sclera. It is found in approximately 73% of normal eyes and, by itself, is a poor indicator of glaucoma [195]. However, the β zone, which is often referred to as a “glaucomatous halo”, has been reported to be associated with glaucoma [194], myopia [196], dropout of superficial and deep parapapillary vessels [197], and visual field progression [198]. A large β zone has been associated with more rapid visual field progression [199].

The γ zone is characterized by the absence of the Bruch membrane and retinal pigment epithelium and is associated with long axial length and MvD [200]. Several reports suspect increased vulnerability of the retinal NFL in eyes with a wide γ zone [77]; however, other studies have reported that the γ zone is not strongly associated with glaucoma [201]. Formation of the γ zone is associated with a temporal shift of the Bruch membrane opening.

The δ zone represents elongation and thinning of the peripapillary scleral flange. It is defined as the area between the dura mater–sclera merging line and Elschnig’s scleral ring. The dura mater–sclera merging line is demarcated by the Zinn–Haller arterial circle [194].

### 10.4. Parapapillary Scleral Ridge and Abnormalities at the Optic Disc Margin That May Affect RNFLDs in Myopic Eyes

As mentioned previously in Section 4, a ridge or localized elevation of the peripapillary sclera may be observed in highly myopic eyes [79,80], and some of these cases are associated with dome-shaped macula [81]. Localized elevation of the collagenous sclera can cause bending of the overlying retinal NFL, potentially leading to axonal damage. In addition, overhang of border tissues beyond the clinical optic disc margin may also contribute to RNFL damage in myopic eyes [20].

## 11. Position of Vascular Trunk

Large vessels within the optic disc are accompanied by supporting tissue and may protect adjacent nerve fibers from glaucomatous damage [202]. When the position of the central vascular trunk shifts nasally or inferiorly, the NFL on the opposite side of the optic disc may become more vulnerable to glaucomatous injury [194]. Accordingly, the intradiscal location of large vessels is considered one of the factors influencing regional vulnerability of the retinal NFL to glaucomatous damage [203,204]. In myopic eyes, optic disc tilting, rotation, and enlargement may influence the location of retinal vessel trunk. There is a considerable interindividual variability in position of vascular trunk among myopic eyes, which makes it difficult to define a consistent or characteristic pattern.

## 12. Retinal Nerve Fiber Layer in Myopic Eyes

### 12.1. Cleavage of the Retinal Nerve Fiber Layer

**Figure 5:**
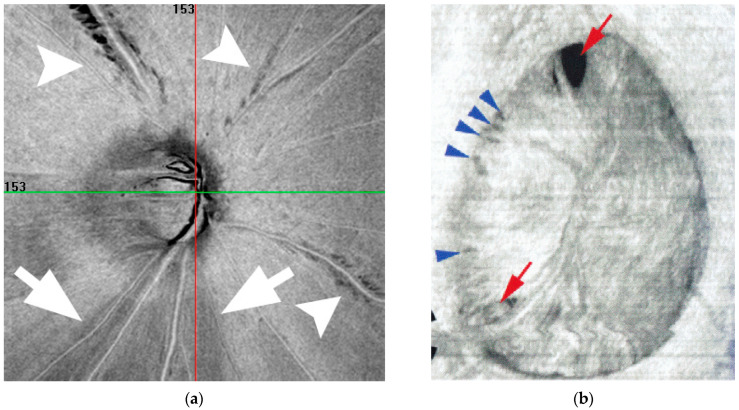
(**a**): Cleavage of RNFL in eyes with high myopia. This is a representative case demonstrating “cleavage” of the retinal NFL in a highly myopic glaucoma eye. A sharply demarcated margin of the cleaved retinal NFL (white arrowheads) contrasts with the blurred margins typically observed in glaucomatous retinal nerve finer layer defects (white arrows). Reproduced with permission from Chihara E [206]. (**b**): Lamina cribrosa (LC) defects in eyes with high myopia. Ectasia of the LC may result in LC defects (red arrows) and pit-like structures (blue arrowheads) in eyes with high myopia. Reproduced with permission from Ohno-Matsui, K. et al. [166].

When two-dimensional ectasia of the posterior pole occurs in myopic eyes, dehiscence of the retinal NFL, referred to as “cleavage”, may develop [205,206,207] (Figure 5a). This finding suggests a mismatch between the fixed number of retinal nerve fiber fibers and the expanded surface area of the ocular wall in myopic eyes.

Retinal NFL cleavage may also be observed in eyes with epiretinal membrane [208] or localized vitreoretinal traction [209]. Such ectasia-associated deformation of ocular tissue may also contribute to the formation of LCDs in highly myopic eyes [166] (Figure 5b). These morphological changes are useful for understanding the mechanical stress imposed on nerve fibers by axial elongation in myopia.

### 12.2. Peripapillary Hyperreflective Ovoid Mass-like Structures (PHOMSs)

PHOMS is a hyperreflective mass-like lesion in the peripapillary region. The pathogenesis of PHOMSs is suspected as herniation of distended axons into the peripapillary retina. PHOMSs are associated with myopia and optic disc tilting [210] and had been diagnosed as buried optic disc drusen or pseudo-papilledema in old years. Although PHOMSs are generally not associated with reduced visual acuity or visual field loss [211], they may lead to retinal NFL thinning [212,213] and enlargement of the blind spot [214]. A wider scleral canal diameter has been reported to be significantly associated with the presence of PHOMSs [215].

## 13. Vascular Anomaly and Vulnerability of the Retinal Nerve Fiber Layer

### 13.1. Microvasculature Dropout (MvD)

Insufficient blood supply to prelaminar or peripapillary regions of the optic disc had long been suspected as contributing factors of retinal NFL damage in glaucoma. Especially, fluctuations in ocular blood flow may trigger a cascade of events involving liberation of cytokines that ultimately impair the retinal NFL [216,217]. Numerous studies have reported a positive association between reduced vascular supply and the presence of VFDs or retinal NFL thinning [218,219,220]. Choroidal-layer MvD is associated with beta-parapapillary atrophy [197], and its enlargement has been correlated with both the severity and progression of RNFL thinning [221,222,223,224,225,226,227]. Furthermore, the topographic distribution of MvD corresponds closely to the location of VFD [228]. In myopic eyes, parapapillary choroidal MvD is significantly associated with central VFDs [229]. Notably, MvD may demonstrate superior diagnostic performance for detecting glaucoma in highly myopic eyes compared with peripapillary RNFL thickness or macular ganglion cell-inner plexiform layer measurements [230].

### 13.2. Choroidal Thickness

The choroid is thin in myopic eyes [82,175,182,188,231]. Reduced peripapillary choroidal thickness may predispose to the development of parapapillary MvD and increase the vulnerability of the retinal NFL in corresponding regions.

### 13.3. Macular Capillary Density

Macular capillary density is reduced and the foveal avascular zone is enlarged in glaucomatous eyes [232,233], whereas the foveal avascular zone has been reported to decrease after glaucoma surgery [234].

## 14. Differentiation of MON and GON

As discussed above, two distinct mechanisms, which were named MON and GON, may underlie glaucoma-like RNFLDs in myopic eyes. The clinical course and response to treatment of these two appear to differ between two entities; therefore, distinguishing MON from GON is clinically important. However, such differentiation is challenging. In highly myopic eyes, the optic disc is frequently enlarged and pale, with a shallow and large cup [177]. Consequently, the spatial contrast between the rim and cup floor is reduced, making delineation of the cup boundary difficult. Moreover, RNFLDs in eyes with high myopia and glaucoma are often diffuse, further complicating discrimination between GON and non-glaucomatous myopic RNFLD challenges. Although differentiation between MON and GON based on ophthalmoscopic findings or OCT findings may be difficult, the clinical characteristics of these two entities differ. The following table may be helpful in summarizing and understanding the distinct clinical features of MON and GON (Table 1).

## 15. Conclusions

Growing evidence suggests that both myopic optic neuropathy (MON) and glaucomatous optic neuropathy (GON) contribute to optic nerve damage in eyes with glaucoma-like optic nerve atrophy. Experimental studies and clinical observations support the concept that elongation of the papillomacular bundle in highly myopic eyes is associated with the development of central VFDs. In addition, deformation of the optic disc and peripapillary structures influences the distribution and pattern of RNFLDs. MON appears to be less dependent on IOP, may progress predominantly during earlier adulthood, and tends to stabilize with aging. The concept of MON may help explain the relatively stable VFDs observed in older patients with myopia who exhibit features of NTG. Recognition of the overlapping and interacting contributions of MON and GON is essential for understanding the pathophysiology of optic nerve damage and for optimizing the diagnosis and management of myopic eyes with glaucomatous features.

## Figures and Tables

**Table 1:** Comparison of clinical characteristics between MON and GON.

	MON	GON
Dependence on IOP	Less dependent on IOP [18]	Apparent IOP dependent
Speed of progression	Slow [18]	Fast
VF Progression associated with age	Progress mainly before age 50 and tends not to progress after 50 [45,46,47].	Older age is a risk factor for progression [48,49,51]
Association with myopia	High [110]	Moderate
Pattern of visual field defects	Atypical patterns, enlarged blind spot, central visual field defects [7]	Bjerrum scotoma, nasal step, paracentral scotoma, and diffuse loss
Papillomacular bundle length	Long [15]	Variable
Deformation of disc	γ-zone commonly present [7,110]	Variable

MON: myopic optic neuropathy; GON: glaucomatous optic neuropathy, IOP: intraocular pressure; VF: visual field.

## Data Availability

This is a review article, and referred publications are available from PubMed Central.

## Abbreviations

The following abbreviations are used in this manuscript:

MON	Myopic optic neuropathy
GON	Glaucomatous optic neuropathy
VF	Visual field
VFD	Visual field defect
BMO	Bruch’s membrane opening
POAG	Primary open angle glaucoma
OAG	Open angle glaucoma
NTG	Normal tension glaucoma
LC	Lamina cribrosa
LCD	Lamina cribrosa defect
ICC	Intrachoroidal cavitation
NFL	Nerve fiber layer
RNFLD	Retinal nerve fiber layer defect
IOP	Intraocular pressure
MvD	Microvascular dropout
pMvD	Parapapillary microvascular dropout
PPA	Parapapillary choroidal atrophy
ONH	Optic nerve head
PHOMS	Peripapillary hyperreflective ovoid mass-like structures
OCT	Optical coherence tomography
